# Nested PCR detection of *JC polyomavirus* large T-antigen in prostate cancer tissues: a case–control analysis in a Sudanese population

**DOI:** 10.1186/s43046-025-00313-y

**Published:** 2025-09-01

**Authors:** Maria Ahmed Mohamed Higair, Babbiker Mohammed Taher Gorish, Sana Eltahir Abdallah

**Affiliations:** 1 Institute of Medical Research, Al-Neelain University, Sudan, Khartoum, Sudan; 2 Department of Microbiology, College of Medical Laboratory Science, Omdurman Islamic University, Omdurman, Sudan; 3 Biofuels Institute, School of Emergency Management, School of Environmental and Safety Engineering,, Jiangsu University, Zhenjiang, China; 4 Department of Pathology, Faculty of Medicine,, Al-Neelain University, Khartoum, Sudan

**Keywords:** *JC polyomavirus*, Prostate cancer, Benign prostate hyperplasia, Large T antigen

## Abstract

**Background:**

The potential involvement of *JC polyomavirus* (*JCPyV*) in prostate cancer (PCa) remains a subject of debate, as existing in vitro studies have produced conflicting results. Understanding the viral oncogenic mechanisms underlying prostate cancer could offer valuable insights into its etiology. This study aimed to explore the association between *JCPyV* infection and prostate cancer by detecting the viral large T-antigen gene in prostate tissue specimens.

**Methods:**

A case–control study was conducted from February 2022 to March 2023, including 100 participants: 50 diagnosed with prostate cancer (cases) and 50 with benign prostatic hyperplasia (BPH) as controls. Formalin-fixed paraffin-embedded (FFPE) prostate tissue samples were collected from all participants. Nested polymerase chain reaction (PCR) was employed to detect *JCPyV* large T-antigen DNA using specific primers. Demographic and clinical data were obtained via a structured questionnaire. Statistical analysis was carried out using SPSS version 20, and associations between *JCPyV* presence and prostate cancer were analyzed using logistic regression.

**Results:**

The mean age of the prostate cancer group was 67.5 ± 10.9 years, compared to 70.9 ± 8.9 years in the control group. *JCPyV* large T-antigen DNA was detected in 29 out of 50 (58%) prostate cancer cases, compared to 19 out of 50 (38%) controls (*P* = 0.045; odds ratio = 1.45; 95% confidence interval: 1.011 to 5.019). Within the prostate cancer group, patients testing positive for the *JCPyV* T-antigen had a mean age of 73.3 ± 8.7 years, significantly higher than T-antigen-negative patients, whose mean age was 67.0 ± 8.3 years (*P* = 0.029).

**Conclusion:**

The prevalence of *JCPyV* large T-antigen gene was significantly higher in prostate cancer patients than in individuals with benign prostatic hyperplasia. These findings suggest that *JCPyV* infection may be linked to an increased risk of prostate cancer, reinforcing prior studies that imply a potential oncogenic role for the virus in prostate carcinogenesis. Further investigations are necessary to elucidate the molecular mechanisms driving this association and its potential clinical implications.

## Background

Prostate cancer is a highly prevalent malignancy affecting the prostate gland, an organ responsible for the production of seminal fluid and the transport of sperm [1]. In its early stages, prostate cancer is often asymptomatic and may not require immediate intervention. Common symptoms, when present, include nocturia, increased urinary frequency, and difficulty urinating. In advanced stages, patients may experience bladder incontinence and back pain due to metastasis [2]. Globally, prostate cancer is the second most frequently diagnosed cancer, accounting for approximately 1.41 million new cases and 375,000 deaths each year [3, 4]. The burden of disease is particularly high in Europe, which contributes 22.8% of global cases and 19.6% of related deaths, and in the Americas, where 29% of cases and 14% of deaths occur. However, Asia and Africa exhibit the highest case fatality rates and proportions of cancer-related deaths [5, 6]. The etiology of prostate cancer is multifactorial, with established risk factors including advanced age, ethnicity, family history, and an interplay between genetic predispositions and environmental influences [4]. Emerging research has also implicated chronic inflammation, possibly caused by infections, as a contributing factor in prostate carcinogenesis [7].

The John Cunningham polyomavirus (*JCpyV*) is a member of the polyomavirus family, characterized by a 5-kilobase (kb) circular double-stranded DNA genome [7]. First identified in 1971, *JCpyV* has been linked to a range of neurological and non-neurological conditions, including medulloblastoma, glioblastoma multiforme, and progressive multifocal leukoencephalopathy (PML). Serological studies indicate that 70–80% of the global population is asymptomatically infected with *JCpyV* [8–10]. As *JCpyV* is highly species-specific and infects only humans, studying its pathogenesis is challenging due to the absence of suitable animal models [10]. Upon primary infection, *JCpyV* targets tonsillar tissue, but curiously, it is not detected in saliva. The virus subsequently disseminates to the kidneys and bone marrow B cells, establishing a persistent latent infection in immunocompetent individuals [11, 12]. In cases of severe immunosuppression, *JCpyV* can reactivate and invade the central nervous system (CNS), where it may cause PML, a devastating demyelinating disorder of glial cells [10–12]. Alternatively, *JCpyV* may establish latency within the CNS and reactivate under specific conditions. *JCpyV* is known to infect astrocytes and oligodendrocytes, which are crucial for the production of myelin. Upon infection, these glial cells undergo cytolysis, leading to progressive demyelination and manifesting in characteristic symptoms of PML, including cognitive impairment, motor dysfunction, speech difficulties, and vision disturbances [10–12]. Additionally, *JCpyV* replication in cerebellar neurons can result in granule cell neuronopathy, manifesting as ataxia, muscle incoordination, and progressive cerebellar atrophy, further illustrating the virus's profound impact on neural function [12].

*JCpyV*, as a DNA virus, hijacks the host’s DNA replication machinery to drive its replication. The viral large T antigen (T Ag) plays a pivotal role in this process by manipulating the host cell cycle. T Ag binds to and inactivates tumor suppressor proteins such as Rb, p107, p130, and p53, which are key regulators of cell division [14]. Through its interaction with Rb, T Ag disrupts the Rb–E2F pathway, promoting uncontrolled cyclin-dependent kinase activity and preventing cells from exiting the cell cycle [14, 15]. The C-terminal domain of T Ag interacts with p53, inhibiting its tumor-suppressive and pro-apoptotic functions, thereby further promoting unchecked cellular proliferation. In vitro studies have demonstrated the oncogenic potential of *JCpyV* T Ag, showing that its expression in cultured cells induces cellular transformation features, including multinucleation, increased doubling time, anchorage-independent growth, and tumor formation in nude mice [14]. Despite these findings, the relationship between *JCpyV* and human cancers remains inconclusive. Although *JCpyV* genomic sequences and viral proteins have been detected in various tumor tissues, a definitive causal link between *JCpyV* and specific cancers, including prostate cancer, has not been firmly established. Some studies report significant levels of *JCpyV* DNA in prostate tissues, suggesting a potential association with prostate cancer development [5, 13–15]. However, other studies have failed to detect a significant difference in *JCpyV* presence between cancerous and non-cancerous prostate tissues [16]. These contradictory findings underscore the need for further investigation into the possible oncogenic role of *JCpyV* in prostate cancer.

Given the detection of *JCpyV* DNA and oncogenic proteins in prostate tumors, it is reasonable to hypothesize that *JCpyV* may act as a co-factor in prostate tumorigenesis. Environmental and social factors, which are continually evolving, may also play a role in modulating viral oncogenesis and disease progression. This study aims to investigate the potential association between *JCpyV* infection and prostate cancer through the detection of *JCpyV* DNA in prostate tissue samples.

## Material and method

### Study setting and population

A descriptive case–control study was conducted from February 2022 to March 2023 across multiple hospitals in Khartoum, Sudan. The study adhered to the STROBE guidelines for case–control studies [17]. Fifty formalin-fixed, paraffin-embedded tissue samples from histologically confirmed cases of prostate cancer were selected as the case group. In contrast, the control group consisted of fifty tissue samples from benign prostatic hyperplasia (BPH) patients, similarly fixed in formalin and embedded in paraffin, and confirmed to be free of malignancy. The control group was age- and socioeconomically matched with the case group. Critically, none of the study participants were undergoing any medical treatment at the time of sample collection, ensuring a baseline assessment.

### Inclusion and exclusion criteria

Patients with histologically confirmed prostate cancer who had not received prior treatment, including surgery, hormonal therapy, or radiation therapy for prostate cancer, were included in this study. Patients exhibiting atypical prostate features, hemorrhagic cystitis, *polyomavirus*-associated kidney disease, or those under treatment for prostate cancer or polyomavirus infection were excluded. These stringent criteria aimed to isolate the impact of *JCPyV* infection by eliminating confounding variables.

### Sample collection

Twenty-five milligrams of formalin-fixed, paraffin-embedded prostate tumor tissue were obtained from residual samples collected during the initial diagnostic biopsy of each patient’s case. Similarly, 25 mg of benign prostatic hyperplasia tissue samples was used as controls. The tissue samples were stored at − 20 °C until analysis. Additional demographic and clinical information, such as age and prostate cancer grade, was extracted from hospital records.

### Molecular analysis and examination

For molecular analysis, approximately 25 mg of each tissue specimen was treated with lysis buffer containing 20 µl proteinase K (200 mg/ml) and 5 µl RNAase A. DNA extraction was performed using the DNeasy® Tissue Kit (Intron Biotechnology), and the DNA samples were stored at − 80 °C until further analysis. DNA purity was assessed using a Nanodrop spectrophotometer. To detect *JCPyV* DNA, nested PCR was performed with three specific primers: T1 3049–3069 (5′ TGGCCTGTAAAGTTCTAGGCA 3′), T2 3229–3207 (5′ GCAGAGTCAAGGGATTTACCTTC 3′), and T3 3193–3171 (5′ AGCAACCTTGATTGCTTAAGAGA 3′), which specifically target the *JCPyV* Maad-1 strain, as previously described [18]. The first round of PCR utilized primers T1 and T2 to amplify a 200 bp DNA fragment, with a reaction mixture containing 1 × PCR premix (Intron Technologies), 20 pmol of each primer, and 10 ng of template DNA in a total volume of 20 µl. The thermocycler was set for an initial denaturation at 94 °C for 5 min, followed by 30 cycles of 95 °C for 30 s, 55 °C for 30 s, and 72 °C for 30 s. In the second PCR round, 2 µl of the amplified product was used as the template for further amplification with T2 and T3 primers, targeting a 145 bp fragment under identical conditions. PCR products were separated via 2.5% agarose gel electrophoresis, stained with ethidium bromide (0.5 µg/ml), and visualized under UV light for analysis. Digital images of the gels were captured for documentation. To prevent contamination, all PCR reactions were prepared in PCR hoods using aerosol barrier tips, with strict adherence to rigorous contamination control measures, including dedicated reagents, frequent equipment sterilization, and the use of separate workstations for pre- and post-PCR processes [19]. Negative controls without template DNA were included for each primer set. If any negative control tested positive, the entire batch of PCR reactions was discarded.

### Data collection and statistical analysis

Data were collected using a validated, pre-approved questionnaire. Statistical analysis was performed using the Statistical Package for Social Sciences (SPSS) version 20 (IBM, Armonk, NY). A *p*-value ≤ 0.05 was considered statistically significant. Results were presented through a combination of tables and graphical visualizations to enhance clarity and interpretation. Additionally, a chi-square test was conducted to explore potential associations between *JCPyV* and malignant transformations in prostate tissue.

### Ethical considerations

This study was conducted in accordance with the Declaration of Helsinki. Ethical approval was granted by Al-Neelain University (Approval number: NU-23–11-2019). Written informed consent was obtained from all participants prior to their inclusion in the study.

## Results

**Fig. 1:**
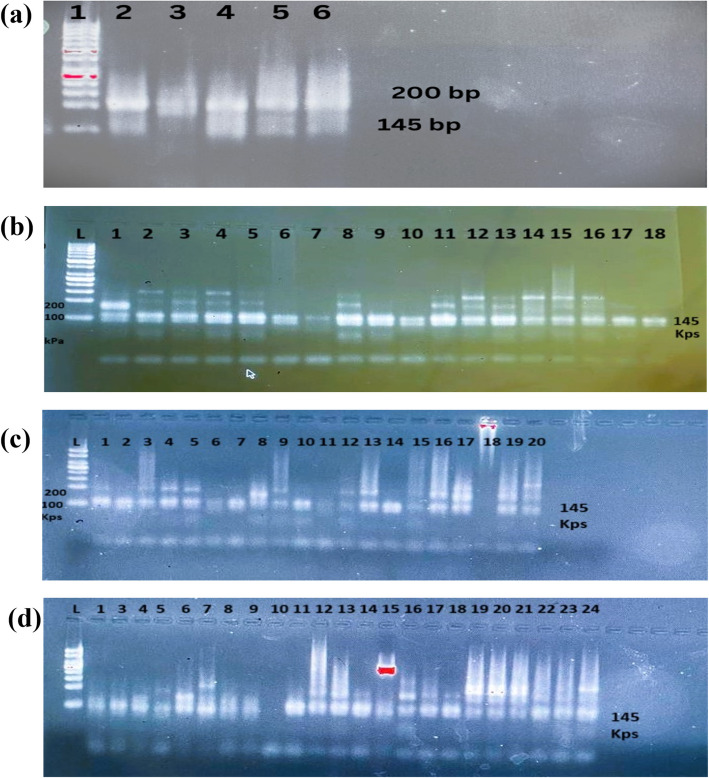
Electrophoretic analysis of nested PCR products targeting the *JCPyV* large T-antigen gene. PCR products were separated on a 2.5% agarose gel, stained with ethidium bromide, and visualized under UV light. **a** Lane 1: 100 bp molecular weight marker; Lane 2: positive control; Lane 3: negative control (distilled water); Lanes 4–6: positive samples. **b**, **c**, **d** Positive controls (B1, C5, D6) show a 145 bp band, while negative controls (B18, C18, D10) show no bands. Positive samples (B3, B4, B5, B8, B11, B12, B13, C4, C8, C13, C16, C17, C19, C20, D12, D13, D16) exhibit a 145 bp band, indicating the presence of *JCPyV* DNA

This study evaluated a total of 100 prostate specimens, comprising 50 benign and 50 malignant tissue samples. The mean age of the prostate cancer cases was 67.52 ± 10.9 years, which, although slightly lower, did not differ significantly from the control group's mean age of 70.88 ± 8.9 years (*P*-value = 0.102). Prostate cancer grades were determined using a standardized cancer grading system [20], revealing that 30% (15/50) of the cases were classified as Grade II and 70% (35/50) as Grade III, reflecting a higher prevalence of advanced-stage tumors in the study population. The detection of *JCPyV* DNA through conventional PCR showed a significant difference between the cancer and control groups. *JCPyV* large T-antigen was detected in 60.4% (29/50) of the cancer cases. In comparison, only 39.6% (19/50) of the benign control samples tested positive for the viral DNA, indicating a statistically significant association (*P*-value = 0.045). The odds ratio of 1.45 (95% CI, 1.0114 to 5.0192) suggests that the presence of *JCPyV* is associated with an increased likelihood of prostate cancer development [Table 1, Fig. 1].

**Table 1:** Comparison between cases and controls in the result of the *JCpyV*-PCR assay

Study participant	Result of *JCpyV*- PCR assay		*P*-value	Odd ratio	95% CI
	Positive	Negative			
Control	19 39.6%	31 59.6%	0.045	1.45	1.0114 to 5.0192
Case	29 60.4%	21 40.4%			
Total	48 100.0%	52 100.0%

A notable finding is the significant disparity in age between *JCPyV*-positive and *JCPyV*-negative prostate cancer cases. The mean age of *JCPyV*-positive cases was 73.2 ± 8.7 years, markedly higher than that of *JCPyV*-negative cases (67.0 ± 8.3 years), with a *P*-value of 0.029 [Table 2]. This suggests that older individuals might be more vulnerable to *JCPyV* infection in the context of prostate cancer, raising the possibility that age-related immunological shifts could play a role in viral oncogenesis. When examining cancer grade, both *JCPyV*-positive and *JCPyV*-negative cases were predominantly Grade III. However, no significant correlation was observed between cancer grade and *JCPyV* presence (*P*-value = 0.662), suggesting that viral infection does not necessarily drive tumor aggressiveness or disease advancement [Table 3]. This finding implies that while *JCPyV* might contribute to prostate cancer initiation, its influence on tumor progression remains uncertain and warrants further investigation. Furthermore, the analysis revealed no significant association between marital status and prostate cancer risk in the context of *JCPyV* infection. The majority of patients were monogamous, and this factor did not confound the observed relationship between *JCPyV* and prostate cancer [Table 3].

**Table 2:** Comparison between *JCPyV*-positive and *JCPyV*-negative cases according to their age means

Study variable	Result of PCR	*N*	Mean	Std. Deviation	*P*-value
Age/years	Positive	29	73.2	8.7	0.029
	Negative	21	67.0	8.3

**Table 3:** Comparison between *JCPyV*-positive and* JCPyV*-negative cases according to their cancer grade and marital status

Cancer grade	Result of PCR		*P*-value
	Positive	Negative	
Grade II	8 27.6%	7 33.3%	0.662
Grade III	21 72.4%	14 66.7%	
Marital status			
Multiple spouses	9 30.0%	7 35.0%	0.253
Single spouse	19 63.3%	12 60.0%	
Unmarried	2 6.4%	1 5.0%	

## Discussion

This study examined the prevalence of *JCPyV* infection in prostate cancer tissues among Sudanese patients between February 2022 and March 2023. The findings revealed that *JCPyV* DNA was present in 60.4% (29/50) of malignant prostate specimens, while 39.6% (19/50) of benign prostatic hyperplasia samples tested positive. The detection of *JCPyV* in both cancerous and benign prostate tissues suggests a widespread presence of the virus, with prostate cancer tissues showing a greater susceptibility to *JCPyV* infection. This supports the hypothesis of a potential association between *JCPyV* and prostate cancer development.

Our study also explored the relationship between clinical characteristics and the presence of *JCPyV* in prostate cancer and benign prostatic hyperplasia patients. Notably, the grade of cancer did not have a significant impact on the detection of *JCPyV*, indicating that the viral infection may not be closely linked to tumor progression or severity. Additionally, no significant differences were found in age between patients with prostate cancer and benign prostatic hyperplasia. This is consistent with previous findings by Anzivio et al., who reported *JCPyV* DNA in 62.5% of prostate cancer patients [15], and Zambrano et al., who found *JCPyV* in 50% of prostate cancer patients, either in tissue samples or urine collections [21]. In contrast, our findings are at odds with those of Gorish et al., who found *JCPyV* DNA in only 23.6% of prostate cancer samples [5]. Additionally, Martinez-Fierro et al. reported no *JCPyV* sequences in any of the 55 prostate cancer tissues they examined [22].

These discrepancies highlight the need for more comprehensive research on the role of *JCPyV* in prostate cancer. Our findings add to the growing body of evidence suggesting that *JCPyV* infection may contribute to the development of prostate cancer. However, conflicting data from other studies warrant further investigation into the precise role of the virus in oncogenesis. Delbue et al. [23] pointed out key characteristics of *JCPyV* that support its potential oncogenicity: the virus is commonly acquired in childhood, remains latent in the host, and possesses oncogenic features capable of disrupting the cell cycle and promoting malignant transformation. *JCPyV* sequences have been identified in various human malignancies, further supporting its role in cancer development. However, detection rates in urine and blood samples remain lower, suggesting that *JCPyV* may initially colonize the urinary tract before invading prostate cells, where it may interfere with apoptosis through interactions with tumor suppressor proteins like p53, mediated by the viral large T antigen.

Emerging research indicates that exosomal circular RNAs (circRNAs) are pivotal in tumor development, particularly in prostate cancer. These regulatory molecules affect gene expression, promote the survival of cancer cells, and may play a role in resistance to chemotherapy. Given their significant influence on prostate cancer biology, exploring whether *JCPyV* infection alters circRNA expression could yield valuable insights into virus-related oncogenesis [25]. Although the detection of *JCPyV* in prostate cancer tissues does not establish a direct causal relationship, its potential oncogenic properties merit further exploration. Future studies should aim to clarify the molecular interactions between *JCPyV* and critical oncogenic pathways, especially regarding its possible effects on circRNAs in the context of prostate cancer progression. Moreover, *JCPyV* infection might aid in immune evasion by affecting immune checkpoint pathways like PD-1/PD-L1, which could lead to new therapeutic strategies [25]. Our research underscores the pressing need for thorough molecular and epidemiological investigations to define the role of *JCPyV* in prostate cancer. Examining circRNA biomarkers in prostate tumor tissues and exosomal pathways could greatly enhance early detection methods and enrich our understanding of viral-induced oncogenesis [26]. Additionally, the relationship between *JCPyV* infection and the tumor-bone microenvironment in castration-resistant prostate cancer is a promising area for further study [27]. Gaining insights into how viral infection impacts these interactions could pave the way for innovative targeted therapies.

One notable observation was that the mean age of *JCPyV*-positive cases was significantly higher (73.2 ± 8.7 years) compared to *JCPyV*-negative cases (67.0 ± 8.3 years), consistent with prior studies conducted in Sudan [6]. Although those studies focused on *BK polyomavirus* (*BKV*), the age-related findings are similar, suggesting that older individuals may be more susceptible to viral carcinogenesis. The higher frequency of viral antigens in older patients supports the hypothesis that aging may enhance the virus’s oncogenic potential, possibly through age-related changes in immune response or cellular susceptibility to viral transformation. This hypothesis should be further explored in future research to determine how aging, alongside viral factors, may contribute to prostate cancer development [28].

It is essential to acknowledge that our research did not explore additional potential risk factors, including alcohol intake, workplace exposures, dietary patterns, familial cancer history, or cadmium exposure. Prior research has suggested that these variables did not have a significant impact on the detection of *JCPyV* or *BKV* in prostate tissue samples [5, 6]. Consequently, although there seems to be a correlation between *JCPyV* infection and the risk of prostate cancer, further investigations are necessary to assess the role of environmental and lifestyle factors in potentially enhancing oncogenic processes.

## Conclusion

Our findings highlight a significant link between *JCPyV* infection and an increased risk of developing prostate cancer, with notably higher levels of the viral large T antigen detected in cancerous tissues compared to controls. These results align with previous studies, reinforcing the hypothesis that *JCPyV* may play a role in prostate cancer development. However, further research is essential to elucidate the precise mechanisms through which *JCPyV* contributes to prostate carcinogenesis and to examine the interplay of additional risk factors.

## Data Availability

The datasets used and/or analysed during the current study are available from the corresponding author on reasonable request.
